# From Measurement Failure to Privacy Infrastructure: Reframing Contact Tracing Governance for the Next Pandemic

**DOI:** 10.2196/99645

**Published:** 2026-10-05

**Authors:** Yusaku Fujii

**Affiliations:** 1 Gunma University Kiryu, Gunma Japan

**Keywords:** no measurement, no control, infectious disease control, privacy infrastructure, measurement failure, contact tracing, verifiable record of AI output, VRAIO, contextual integrity, public health surveillance

## Abstract

No measurement, no understanding; no understanding, no control: this foundational scientific principle was exposed as a public health dysfunction by the COVID-19 pandemic. Transmission chains spread invisibly, and the contact histories, mobility patterns, and biosignals necessary for control were never systematically collected. Although sensors and digital technologies existed, the fundamental reason measurement failed was the absence of privacy infrastructure that would have enabled people to provide data with confidence. This failure had structural reasons. The object of measurement in infectious disease control is not a physical phenomenon but human beings, and measurement therefore enters the core of privacy: contact histories, social relationships, and bodily states. Because greater precision also deepens privacy intrusion, contact-tracing apps faced 2 failures: privacy-centered designs lost epidemiological utility, while utility-centered designs were rejected through public distrust. Neither achieved sufficient measurement. This Viewpoint reframes the problem. Privacy protection is not a constraint that impedes infectious disease control but the enabling condition upon which effective measurement depends. Existing regulations and technical methods have not been designed from this premise and have therefore failed to break the cycle of structural distrust. As an institutional approach to filling this gap, we present VRAIO (verifiable record of AI output), which integrates democratic rule-setting, metadata declaration, third-party verification, tamper-proof ledgers, and violation-deterrence incentives. Once privacy infrastructure is established, this foundational principle can operate freely in infectious disease control for the first time. It will enable high-resolution epidemiology and precision intervention, opening a new path for public health that reconciles infection control with individual autonomy and social freedom without relying on blanket social shutdowns.

## Introduction: No Measurement, No Understanding, No Control

No measurement, no understanding. No understanding, no control. This is a foundational principle running through natural science and scientific technology. From classical mechanics to contemporary AI, this chain admits no exception; it applies equally to infectious disease control.

The COVID-19 pandemic showed how this principle can fail in public health. Contact histories, mobility patterns, and biosignals necessary for control were not systematically collected, and authorities were trying to control a system they could not see. Before the Wuhan mobility restrictions, approximately 86% of all infections were estimated to have spread undocumented, and 79% of documented cases were attributed to these invisible transmission chains [1]. It has also been shown that surveillance recorded deaths while systematically underestimating the true scale of infection [2]. At its core, COVID-19 was a “measurement failure.”

However, this was not a failure of technology. Sensors, algorithms, and communications infrastructure existed; contact-tracing apps were deployed in many countries; and new data streams were available [3]. UK data showed that each 1-percentage-point increase in app adoption reduced infections by approximately 0.8% to 2.3% [4]. What failed was not the availability of measurement tools but the prerequisite that would make measurement acceptable to citizens: privacy protection.

This Viewpoint argues that privacy infrastructure is not a secondary issue attached to epidemiological architecture but the foundational condition for effective measurement and control. The Viewpoint first explains why infectious disease measurement overlaps with privacy, then analyze 2 patterns of failure in contact tracing, present institutional redesign through VRAIO (verifiable record of AI output), and finally discuss the future of infectious disease science once the barriers to measurement are removed.

## Why Infectious Disease Measurement Is a Privacy Problem

The object of measurement in infectious disease control is not a physical phenomenon but human beings, and measurement therefore inevitably enters the core of privacy. Unlike monitoring bridge deterioration or the weather, infectious disease transmission measurement handles behavioral records: who was where, when, and in contact with whom. It discloses social relationships and directly implicates informational self-determination. Moreover, collecting heart rate, body temperature, blood oxygen levels, and similar data through wearables extends measurement into the domain of bodily integrity. The very fact that personally identifiable information, location data, and vaccination records had to be shared with authorities impeded the adoption of contact-tracing apps [5].

This problem is not confined to contact tracing. The moment measurement reaches the body or behavior of an individual, it becomes social measurement. Although this Viewpoint focuses on contact tracing, its thesis—no legitimate social measurement without trust infrastructure—extends to social measurement involving privacy in general.

This point is captured by the concept of contextual integrity. As Nissenbaum [6] argues, the appropriateness of information flow is determined by the norms of the social context in which the information is generated. The contact, behavioral, and biometric data used in infectious disease measurement are generated in health care and public health contexts; diversion to commercial, law enforcement, or surveillance purposes is a privacy violation even if technically feasible [7]. Resistance to contact-tracing apps was inseparable from distrust that data provided for epidemic control might be repurposed. Infectious disease measurement is therefore a political and ethical undertaking that asks not only whether measurement is possible but also whether it is permissible [8].

Here lies a structural asymmetry. In engineering measurement, improved precision is welcomed; in infectious disease measurement, greater precision, granularity, and coverage also deepen privacy intrusion. A structure that mobilizes individual informational sovereignty as a means of infection prevention cannot be resolved by technical ingenuity alone. The World Health Organization (WHO) has also recognized that improving measurement systems for future threats requires trust and cooperation with society [9].

Therefore, a privacy-protection foundation must be built as social and technical infrastructure beyond individual technical improvements and legal rules. Sufficient measurement cannot be achieved without an institutional structure in which each person can concretely trust how their data will be handled. Official assessments share the view that almost every country’s surveillance system has vulnerabilities and that “bold changes” are necessary [10]. However, the debate remains skewed toward measurement infrastructure, while the privacy-protection foundation that would make implementation socially possible has not been systematically addressed.

## Two Patterns of Failure: COVID-19 Contact-Tracing Apps as a Case Study

Contact-tracing apps during the COVID-19 pandemic were a case in which the tension between measurement and privacy became acute. They were deployed in many countries but failed to fulfill their expected role. A prospective evaluation of Australia’s COVIDSafe app found that contacts additionally detected by the app amounted to less than 0.1% of those not identified by conventional tracing, and no significant contribution to the 6-month response was observed [11]. This was not the failure of a particular country or design decision but a structural failure.

That structure is the trade-off between privacy and data utility [12]. Policymakers are drawn in 1 of 2 directions: prioritizing privacy and losing utility, or prioritizing utility and being rejected through public distrust. Either way, sufficient data are not collected, and control is not achieved.

The first pattern is loss of utility through privacy prioritization. The Google Apple Exposure Notification (GAEN) framework collected no location data; only anonymous encrypted identifiers were exchanged between devices, and infection determination was completed on the device. It was strong in terms of privacy protection but could not obtain epidemiologically important information such as place, movement route, and contact context, making secondary contact tracing and identification of superspreader events difficult. As Meijerink et al [13] note, decentralized apps collect no data, making epidemiological evaluation itself difficult. Norway’s Smittestopp version 1 was suspended because of privacy concerns, and version 2 shifted to a GAEN-type design, but epidemiological analytical capability was substantially lost as a result [13].

The second pattern is public distrust caused by insufficient privacy consideration. A national Australian survey found that privacy concerns were the most common reason for nonadoption of COVIDSafe (25%) [14]. Another study similarly identified privacy concerns as the leading reason for nonadoption (31%), followed by distrust of government (18%) [15]. Ho et al [5] showed a mechanism in which concerns about sharing personally identifiable information, location data, and vaccination records, together with government distrust, impeded adoption. In Singapore, the adoption of TraceTogether increased from 38.4% under voluntary uptake to 85.1% following coercive intervention; however, the coercive measures themselves led to a further loss of trust [16]. Even when technical privacy design is appropriate, the subjective distrust that “the government wants to track me” may remain [17]. Distrust is a barrier that fundamentally governs technical effectiveness [18].

Common to both patterns is the fact that the privacy problem cannot be solved by technical improvement alone. Bluetooth ranging accuracy, battery consumption, and the digital divide are subjects for engineering improvement. Privacy, however, requires institutional design, democratic legitimacy, and sustained legal infrastructure.

The failure of contact tracing cannot be attributed solely to privacy concerns. Privacy concerns repeatedly rank high among reasons for nonadoption [14,15], but distrust of government, doubts about usefulness, disparities in smartphone penetration and literacy, and constraints in the public health system also acted independently [5,18]. Infectious disease measurement also consists of multiple data streams—contact apps, clinical records, syndromic surveillance, mobility data, and wastewater epidemiology—all of which directly involve privacy and require trust infrastructure for integration [3,8]. This Viewpoint focuses on privacy infrastructure not because it is the sole cause but because it is a common necessary condition.

Here lies a vicious cycle. If privacy protection is incomplete, distrust persists; if distrust persists, adoption cannot exceed the effective threshold; if uptake is insufficient, utility is not demonstrated; and if utility is not demonstrated, investment in institutional infrastructure is not prioritized. As Bengio et al [19] warned, without appropriate attention to privacy, public trust cannot be obtained, and without trust, broad uptake cannot be achieved. However, this warning has not been sufficiently translated into substantive investment in institutional infrastructure.

## Reframing the Problem: Privacy Protection as the Core

### Existing Approaches and Their Limitations

Efforts to protect privacy have progressed on both social and technical fronts. On the social side, frameworks such as the European Union (EU) General Data Protection Regulation (GDPR) [20], EU AI Act [21], Digital Services Act [22], and the US Children's Online Privacy Protection Act [23] have normatively established purpose limitation, consent, data subject rights, and transparency. Legal demands for privacy protection in health information have also continued to grow [24]. However, these frameworks mainly declare what must not be done; they do not constitute social infrastructure for continuous and verifiable enforcement of violations.

On the technical side, differential privacy [25], decentralized architectures, anonymization and pseudonymization, federated learning, and encrypted communications have been implemented [26,27]. GAEN-type decentralized design is one example. However, the preceding analysis of contact-tracing apps shows that decentralized design does not fundamentally resolve the trade-off between privacy protection and data utility, and it makes epidemiologically necessary information difficult to obtain [12]. More generally, risks of information reconstruction through AI inference remain [26,27], and the black box nature of machine learning models obstructs accountability for internal processes [28,29]. Technical improvements can make risks manageable but cannot remove the barriers to measurement in principle.

Both social and technical approaches are indispensable, but neither is sufficient alone (necessary but insufficient). Their common gap is an institutional foundation that addresses the structural problem: without appropriate attention to privacy, neither trust nor broad participation can be obtained [19]. The fact that nontechnical considerations govern technical effectiveness has not been sufficiently institutionalized [18,30].

The point to reconsider is that privacy protection is not a constraint that impedes infectious disease control. As McGraw et al [24] argue, privacy protection is an enabler: people participate in health information sharing precisely when trust is established. The trade-off between privacy and utility is a limitation of current technologies and institutions, not a principled limit.

The problem to be solved is the institutional establishment of a privacy-protection foundation at the scale of social infrastructure: a structure in which each person can concretely trust how their data will be handled.

Such a structure requires 3 conditions. First, permitted and prohibited outputs must be transparent and predictable to citizens. Second, commitments must be continuously verifiable in real time rather than checked only after the fact. Third, the incentive structure must make violation an irrational choice in advance. Existing regulations and technical methods do not satisfy these 3 conditions simultaneously.

VRAIO is one concrete proposal in this direction [31]. It was originally proposed for AI-connected cameras in public spaces, but its core idea—ex ante control of output—can also be applied to personal data in infectious disease measurement. In both cases, the essence of the problem is whether citizens can trust what their data will be used for.

The point of governance in VRAIO is concentrated not inside the AI system but at output. The data analyzer may handle full-resolution data inside the outbound firewall, but VRAIO governs only the moment when analytical results cross the boundary toward external receivers. Requests themselves are not governed. Even if an output candidate is generated from a nonconforming request, it fails recorder verification, is blocked by the valve, and does not become output. The rules distinguish permitted purpose and content by destination, such as the individual and public health authority (Table 1).

**Table 1:** Receiver-specific rules in contact tracing.

Receivers	Permitted purposes	Permitted content and granularity
General public (including citizens, employers, and insurers)	Situational awareness and risk avoidance	Population-level, nonidentifying information only (eg, regional spread status and high-risk area alerts)
Individuals (data subjects)	Awareness of one’s own exposure or status	Full-resolution information about oneself, accessible only to the individual concerned
Public health planners	Cluster detection, epidemic modeling, and resource allocation	Pseudonymized, aggregated cluster or mesh-level information and no individual-identifying granularity to third parties
Enforcement or notification bodies	Issuing exposure notifications	Only the minimum information necessary for notification, with no broader behavioral history
Health care institutions	Treatment of the specific patient	Only the data of the patient under that institution’s care
Research institutions	Analysis, modeling, and simulation	May drive repeated analysis on the inside-boundary data analyzer and only nonidentifying products (model parameters, aggregate or differentially private statistics, and simulation results) leave the boundary

### VRAIO in Operation: 5 Layers Centered on Output Governance

#### Overview

The design principle of VRAIO is concentrated at a single point: what ultimately affects society is the output of the AI system. VRAIO therefore makes output the primary object of governance. If output can be limited to the purposes and content democratically permitted by society, social accountability can be established without fully elucidating the interior of the data analyzer. The following 5 layers support this point (Figure 1): democratic rule-setting (rules), metadata declaration, formal verification by the recorder, full recording in a tamper-proof ledger, and ex ante destruction of misuse through incentives. However, even if output is governed, the fact that the AI system “knows” the data remains. VRAIO is not a universal remedy but a realistic step toward addressing the greatest barrier.

In a transitional period where social trust remains insufficient, input-stage regulation and approaches oriented toward value formation inside AI [32] may also be useful complements. VRAIO centers governance on outputs not to exclude them but because it has the practical advantage of establishing accountability without waiting for the interior to be clarified.

**Figure 1:**
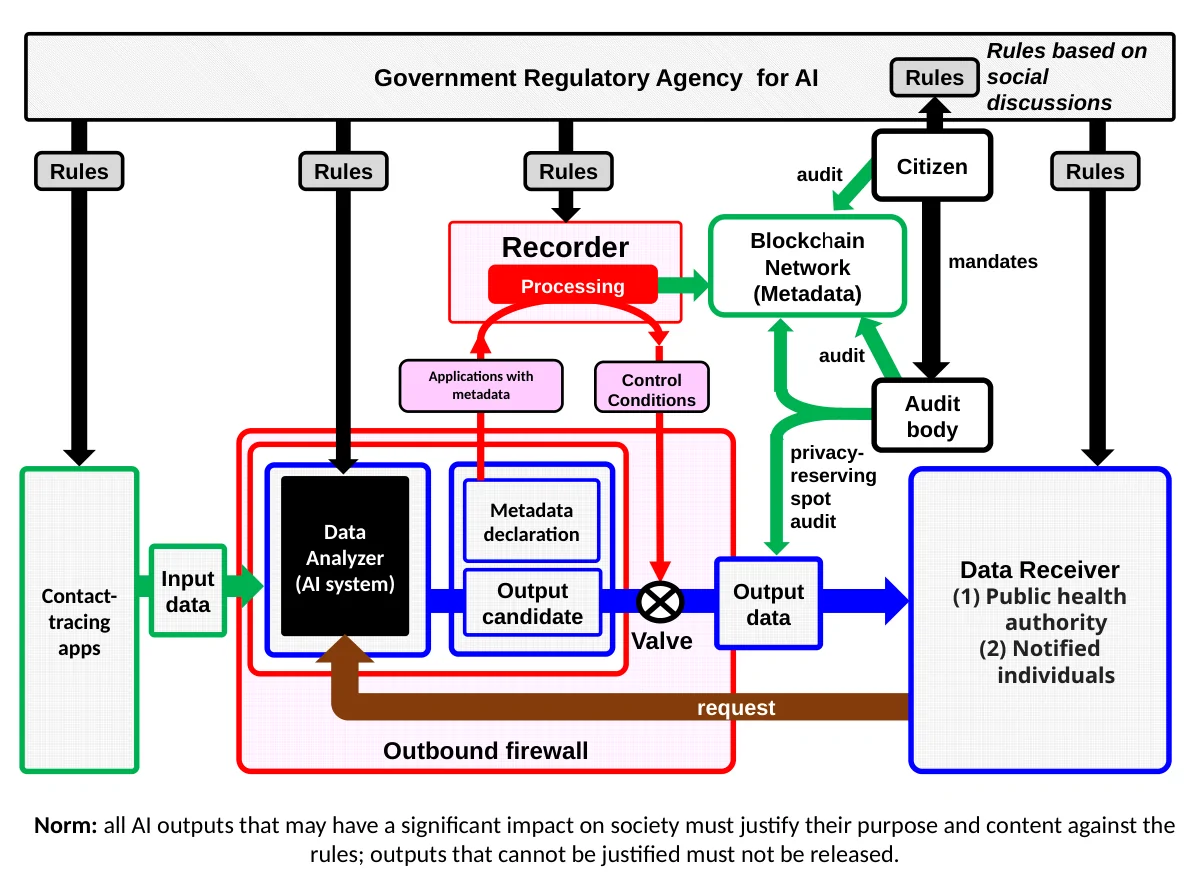
Structure and operation of VRAIO (verifiable record of AI output) applied to contact tracing. The data analyzer is located inside the outbound firewall boundary; any information released across that boundary, including analytical results, is treated as “output” and governed by VRAIO.

#### Democratic Rule-Setting (Rules)

Permitted and prohibited outputs are defined at the level of legislation through democratic processes by the legislature and regulatory authorities. The rules translate obligations under instruments such as the GDPR [20] into machine-readable schemas—field definitions, value domains, and logical conditions for metadata—and are made public, including to operators and the recorder. The essence is to distinguish the purpose and content of output by receiver. The legitimacy of the same information varies with its destination [6,7]. For example, population-level infection status and high-risk area information may be provided broadly at nonidentifying granularity. An individual’s own exposure status may be disclosed to that individual at full resolution, while public health authorities receive only pseudonymized or aggregated information; third-party provision at individual-identifying granularity is not permitted. The correspondence is shown in Table 1.

#### Metadata Declaration

For every output candidate, the data-processing system attaches structured metadata indicating “purpose, content, and receiver”; sends it to the recorder; and receives a determination on whether the output may be released.

#### Formal Verification by the Recorder

The independent third-party recorder does not touch the output content itself; it verifies only whether the declaration formally conforms to the rules. Output that does not pass through the recorder is physically impossible through the outbound firewall (Figure 1), and only conforming output passes the valve. The recorder is independent of operators and regulatory authorities and holds no decryption keys; therefore, it cannot access the content. This design avoids the need to elucidate the black box [28,29] or address the epistemological limitations of internal regulation approaches [33]. Requests are not objects of governance; the governance point is unified at the output stage. The recorder’s own independence is supported by ledger transparency and external audit.

#### Full Recording in a Tamper-Proof Ledger

The declaration, verification result, and determination process are irreversibly recorded in a distributed ledger (the blockchain network in Figure 1). A record infrastructure accessible to audit bodies, independent investigators, researchers, and citizens enables multilayered verification, including verification of the recorder itself.

#### Ex Ante Destruction of Misuse Through Incentives

A false declaration—submitting metadata that differs from the actual output—leaves a trace in the ledger and is therefore easy to discover after the fact, triggering sanctions at a level that threatens the survival of the operating entity. Privacy-preserving spot audits reinforce this (Figure 1). The essence of VRAIO is not to “monitor and catch” but to provide infrastructure that “makes violation an irrational choice in advance” [18]. Trust cannot be obtained through technical design alone [17]; institutional structure complements it.

VRAIO governs the output stage and does not replace privacy-preserving technologies at the collection stage. Differential privacy [25], federated learning [26], and GAEN-type decentralized design [13] complement VRAIO as means of determining output granularity and lowering reidentification risk. VRAIO realizes its value when a verifiable output-governance layer is superimposed on these methods.

The primary aim of VRAIO is not to restrict outputs but to enable analysis, model building, and simulation using full-resolution data within the data analyzer (Figure 1), while releasing only nonidentifying models and aggregate results at a granularity appropriate to each recipient. This enables characterization of infection status; higher-fidelity infection models; and more localized, individualized, and efficient countermeasures while preserving privacy. The purposes and permitted granularities for each receiver are summarized in Table 1.

### Integrative Significance as Privacy-Protection Infrastructure

VRAIO does not replace existing social or technical approaches; it functions as infrastructure that connects them. If rule conformity by output destination is verifiably guaranteed, the collection and computation methods inside the boundary can be selected according to the application. Federated analytics analyzes without aggregating data; secure multiparty computation [34] obtains statistics without disclosing raw data; and trusted research environments [35] allow researchers to analyze full-resolution data within controlled areas and carry out only aggregate results. All of these approaches can be incorporated inside the VRAIO boundary. The GDPR’s purpose limitation principle [20], differential privacy [25], and algorithm auditing [33] also mutually reinforce one another on VRAIO’s verification and enforcement layer. VRAIO is a common layer that guarantees that whatever method is used, the product released to the outside is legitimate.

Trust infrastructure is a necessary condition for infectious disease measurement. Cryptography, distributed ledgers, privacy engineering, and legal-institutional design are all indispensable, but none alone constitutes infrastructure. What is needed is an integrated function that brings them together, verifiably enforces conformity to receiver-specific rules, and deters violations. To the best of our knowledge, VRAIO is currently the only proposal that concretizes this integration as an institutional design. The claim of this Viewpoint, however, is not that VRAIO is the only answer but that research and development should be directed toward integrated infrastructure rather than the mere accumulation of individual technologies.

Some issues remain beyond the scope of this Viewpoint. First is the trustworthiness of the recorder. VRAIO presupposes an independent third-party institution, and its appointment, supervision, and separation of powers belong to the design of audit systems and regulatory institutions. What VRAIO adds is that the recorder’s determinations themselves are recorded in a ledger and made subject to independent verification, as described above. Second, the legal basis for sanctions is left to national legislation, but VRAIO provides an evidentiary foundation: false declarations remain as tamper-proof records. Third, cross-border measurement requires national laws and international coordination. Provision to foreign receivers is also treated as destination-specific output and permitted only within the scope allowed by each country’s law (Table 1).

What, then, becomes possible when the door of privacy protection is opened? This question leads directly to the broader implications of privacy protection for infectious disease science.

## Privacy Protection as the “Door to the Next Stage”

The establishment of privacy protection is not the end point of infectious disease control but the entrance to the next stage.

### From “Constraint” to “Capability-Enabling Condition”

In public health, privacy protection is often regarded as a constraint that impedes stronger infection control. However, as COVID-19 showed, the main cause of measurement failure was not the absence of technology but the absence of an institutional foundation that allowed people to participate with confidence. The question is not “what must be sacrificed to protect privacy” but “what becomes possible precisely because privacy can be protected.”

### The Horizon of Liberated Measurement

If the door of privacy protection opens, many measurement technologies can move toward social implementation. Contact-tracing apps are the paradigmatic case: they deliver sufficient epidemiological value only when adoption exceeds a certain level [36]. The same logic extends to functional expansion. PAPR for Everyone [37], which deploys powered air-purifying respirators as a third axis against airborne infectious disease, and PWS-NET [38], which records wearing status at social scale, measure “compliance with protective behavior.” A powered air-purifying respirator equipped with a differential-pressure sensor can continuously estimate respiratory flow [39], opening a path to continuous measurement of respiratory state. Digital phenotyping may detect presymptomatic changes [40]. Long-term accumulation of routine testing records also depends on voluntary participation once concerns over discrimination and employment disadvantage are removed. Contact, mobility, testing, and biosignal data, when safely linked, can provide high-resolution understanding of the relationship among exposure, infection, and onset. The greatest bottleneck is not technological insufficiency but the absence of trust in privacy protection [8].

### High-Resolution Epidemiology and the Self-Reinforcing Loop

If these data are safely integrated, the resolution of epidemiology changes qualitatively. Beyond conventional spatiotemporal characterization, next-generation measurement may quantitatively explain the conditions under which infection occurs. If risk characterization moves from coarse categories to precise understanding, countermeasures can evolve from uniform implementation to local and selective concentration of resources on high-impact interventions [41]. Better measurement deepens understanding, understanding produces appropriate control, and verification of effects justifies further investment in measurement. When this virtuous cycle is established, infection control becomes an adaptive learning system that links measurement, modeling, localized intervention, and immediate verification. Higher resolution is not an intensification of surveillance but a condition for avoiding unnecessarily broad blanket restrictions.

This virtuous cycle, however, has prerequisites. Measurement failure does not simply mean that no measurement was performed. During the pandemic, reported values were often treated at face value without evaluating temporal biases such as reporting delays, revisions, and backfills, creating the risk that apparent decreases would be mistaken for convergence. To obtain reliable epidemiological signals from heterogeneous surveillance systems, systematic biases must be separated from random variation, and measurements must be handled correctly [42]. For AI output as well, retrieval-grounded evaluation has been presented as a direction for making factuality auditable, supporting the verifiability of VRAIO at the output stage [43].

### Remaining Limitations

Nevertheless, VRAIO-type infrastructure does not completely eliminate the trade-off between privacy and utility. The first challenge is implementation cost. Recorder verification, firewall blocking, and spot audits all become more costly as completeness is pursued; VRAIO therefore adopts probabilistic ex post deterrence rather than full inspection. The second challenge is more fundamental: the legitimacy of output depends on the definition of permitted purpose, but society cannot specify this with perfect precision. Rules are not a one-time completed product; they should be updated as social consensus evolves. What VRAIO guarantees is verifiable conformity to given rules, not the correctness of the rules themselves. Uneven digital access and uneven institutional trust are also matters that the rules must address.

### Conclusions

The principle “no measurement, no understanding; no understanding, no control” has not been adequately realized in public health. The COVID-19 pandemic demonstrated the consequences of this failure on a devastating scale. However, the root cause was not a lack of technology but the failure to institutionalize the prerequisite for measurement: social trust in privacy protection.

This recognition reverses the structure of the problem. Privacy protection is not a constraint on infectious disease control but a capability-enabling condition that makes effective measurement socially possible. Unless this shift is accepted, structural barriers to measurement will remain even if more refined sensors and stricter legal regulations are developed.

The question to solve is clear: can society build, at infrastructure scale, an institutional structure in which citizens can concretely trust how their data will be handled? VRAIO infrastructure is one response to that question, but not the only one. What matters is that research and development in this direction begin before the next pandemic.

If privacy infrastructure is established, the trade-off between stronger surveillance and the loss of freedom can be resolved for the first time on a common foundation that supports both. “No measurement, no understanding; no understanding, no control”: the day this principle begins to operate fully in infectious disease control is within reach, not as a technological matter, but as an institutional one.

## Abbreviations

EU: European Union
GAEN: Google Apple Exposure Notification
GDPR: General Data Protection Regulation
VRAIO: verifiable record of AI output
WHO: World Health Organization

## Data Availability

Data sharing is not applicable to this article as no datasets were generated or analyzed during this study.
